# A dataset exploring urban comfort through novel wearables and environmental surveys

**DOI:** 10.1038/s41597-024-04279-9

**Published:** 2024-12-23

**Authors:** Patrick Chwalek, Sailin Zhong, Nathan Perry, Tianqi Liu, Clayton Miller, Hamed Seiied Alavi, Denis Lalanne, Joseph A. Paradiso

**Affiliations:** 1 MIT Media Lab, Cambridge, MA USA; 2 University of Fribourg, Fribourg, CH Switzerland; 3 Swiss Federal Institute of Technology, Lausanne, CH Switzerland; 4 National University of Singapore, Singapore, SG Singapore; 5 University of Amsterdam, Amsterdam, NL Netherlands

**Keywords:** Electrical and electronic engineering, Psychology and behaviour

## Abstract

This study presents a comprehensive dataset capturing indoor environmental parameters, physiological responses, and subjective perceptions across three global cities. Utilizing wearable sensors, including smart eyeglasses, and a modified Cozie app, environmental and physiological data were collected, along with pre-screening, onboarding, and recurring surveys. Peripheral cues facilitated participant engagement with micro-EMA surveys, minimizing disruption over a 5-day collection period. The dataset offers insights into urban comfort dynamics, highlighting the interplay between environmental conditions, physiological responses, and subjective perceptions. Researchers can utilize this dataset to deepen their understanding of indoor environmental quality and inform the design of healthier built environments. Access to this dataset can advance indoor environmental research and contribute to the creation of more comfortable and sustainable indoor spaces.

## Background & Summary

Indoor environmental health and comfort have received increased attention since the onset of the most recent pandemic. In developed countries, we typically spend over 90% of our time indoors, yet indoor environments exhibit significant variability both between and within buildings^1,2^. Adding complexity, individual comfort is highly subjective, influenced by factors such as local climate, personal preferences, and individual physical and psychological attributes^3,4^. To gain deeper insights into individual exposure and preferences, comprehensive datasets ideally should encompass diverse environmental measurements closely linked to human health and comfort, along with synchronized subjective assessments.

Measuring individuals’ environmental conditions and physiological responses across different contexts poses challenges. While many commercially available sensor-rich systems are readily accessible, they often abstract away pre-processing and system intricacies from the user. Although technologies exist for monitoring physiological parameters (e.g., smartwatches), there is a scarcity of solutions optimized for continuous environmental monitoring throughout the day. The placement of environmental sensors around users is a topic of debate and depends on the specific characteristic being measured^5,6^. For instance, for gases related to human breathing dynamics, proximity to the mouth is optimal, whereas temperature sensors afford more flexibility in placement. However, placing sensors near or on the face may present logistical constraints and discomfort for users, which could impact data accuracy. Additionally, longitudinal studies of environmental exposure should encompass periods spent outside buildings and during commuting, as these factors can vary significantly based on geographical location, local events, and transportation modes^7^.

Various methods exist in the literature and practice for collecting subjective data on users’ comfort and perception of their environments. A recent example is the Cozie iOS and Apple Watch application, offering an open-source approach for collecting survey data in a reliable, longitudinal, and non-intrusive manner^8^. The application incorporates a dynamic micro-ecological momentary assessment (EMA) question flow, capturing users’ location and discomforts, including thermal comfort and noise, with the flexibility to tailor assessments for specific data collection needs.

When seeking accurate sensor measurements to correlate with user perception or exposure (e.g., light levels, temperature, humidity, air quality), smart glasses emerge as an attractive option. Positioned near the eyes, ears, mouth, and nose—critical areas for visual and auditory perception, as well as inhalation and exhalation of contaminants—smart glasses offer promising avenues for data collection. For our study, we utilized the AirSpec Platform, designed for both quantitative and qualitative data collection^9^. Based on a previous smart eyeglass design, AirSpecs have been evaluated for physical comfort and appearance, demonstrating equivalence to other popular commercial smart eyeglass systems^10^.

In this paper, we present our data collection efforts across three geographical using the AirSpecs platform^9^ in conjunction with a custom iOS and Apple Watch application built for AirSpecs with inherited traits from the Cozie app^8^. We outline our data collection methodology, summarize dataset variance, provide access instructions, and offer guidance on utilization.

## Methods

### Experimental design

**Fig. 1:**
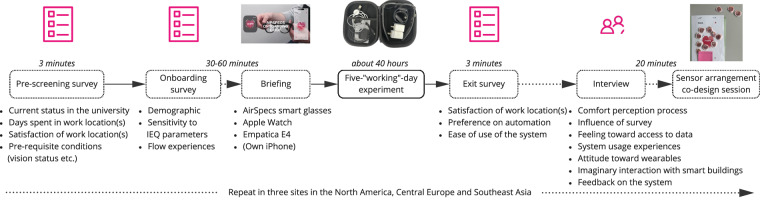
Overview of study design.

**Fig. 2:**
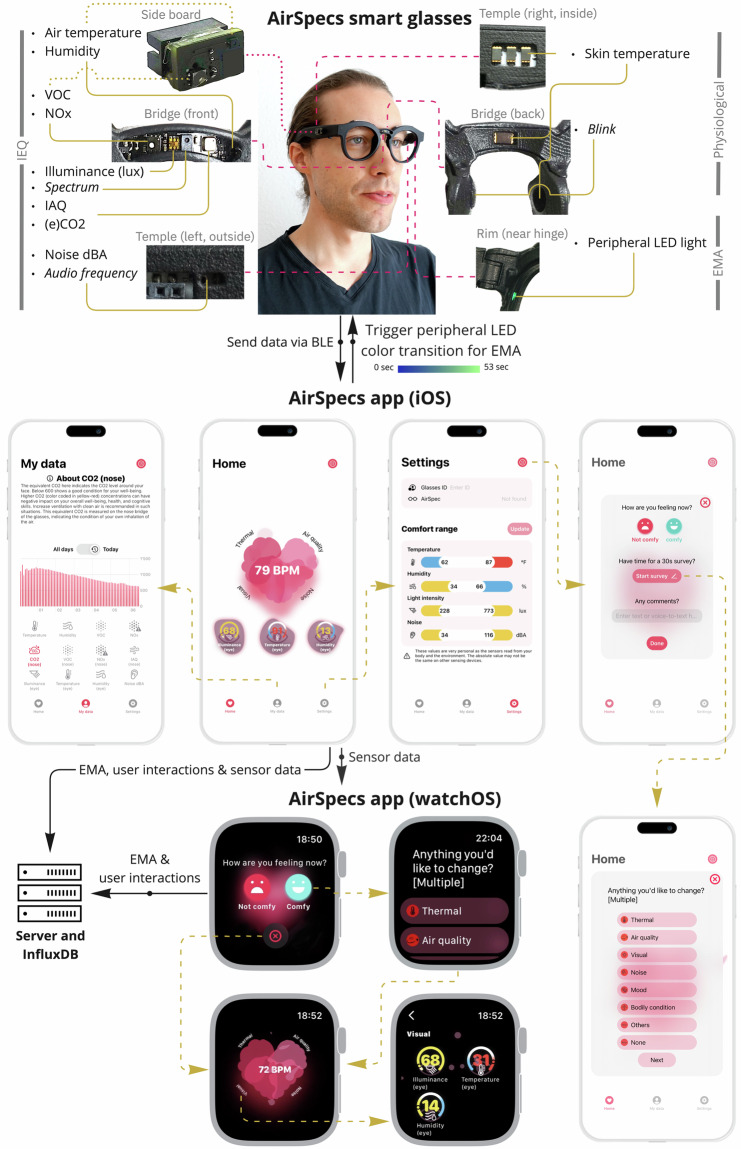
System overview of AirSpecs device and apps. The sensor readings of the device in the top figure (except those in *italic*) are accessible by the user. Most readings are presented in raw form, except that skin temperature data was used to estimate cognitive load. Screenshots of the AirSpecs apps design in iOS and the corresponding watchOS are shown, including home, historical records, settings, and the survey. The watchOS app aims to provide spontaneous information, so we eliminated the historical records and settings screens in its design. Two sets of sensors that measure air temperature, humidity, VOC, and NOx are located in the left temple (on a sideboard) and bridge, considering that measurements near the breathing area and the surrounding environment can differ.

The study encompassed three geographical regions to diversify cultural backgrounds and climatic conditions: Boston, Massachusetts, USA, in March/April 2023 (Site 1); Fribourg, Switzerland, in May/June 2023 (Site 2); and Singapore in June/July 2023 (Site 3). The experiment was approved by the Institutional Review Board (IRB) of each study site, specifically, the Massachusetts Institute of Technology’s IRB (2301000858), the University of Fribourg’s IRB (2023-826-R2), and the National University of Singapore’s IRB (NUS-IRB-2023-135). Ten participants were recruited via lab-wide email advertising at each location, and data collection occurred continuously over five days during working hours. Collection mechanisms involved pre-screening, onboarding, and recurring Ecological Momentary Assessment (EMA) surveys adapted from the Cozie iOS application^8^, Apple Watch, Empatica E4 wristband, AirSpecs^9^, and an exit interview (see Fig. 1). All the participants were invited to an in-person onboarding session where a consent form showing the purpose, study procedures, risks, and potential discomfort were explained to the participants. In addition, a datasheet with a full list of sensor data to be acquired through Empatica E4, Apple Watch/iPhone, and AirSpecs as well as a data storage flow (a simplified version of Fig. 2) were presented. The ethical committees considered this study to have a low level of security risk since (1) the smart glasses did not record dialog;(2) the data through glasses was transmitted in binary format and without source code, it could not be decoded; (3) in all Apps (both AirSpec and Cozie iOS App), participant’s identifiable personal information were not recorded, instead, their data was annotated with participant ID only; (4) the Empatica E4 data was stored locally (without any personal information). With the understanding of this information, the participants were informed that they would be able to withdraw from the experiment at any time and would have the option not to have the Cozie App if they had concerns about data being shared with Apple.

In total, 30 participants were selected, comprising 14 women, 14 men, 1 non-binary/third gender participant, and 1 who preferred not to disclose, aged between 21 and 52 (refer to Table 1). Participants received compensation totaling 150 local currency vouchers (equivalent to $112-172 USD) for the entire study period, including onboarding and interview sessions. Twenty-nine participants used their own iPhones, with eight also utilizing their own Apple Watches. Others were provided with an Apple Watch 7/8 and an iPhone SE (2nd Gen). Approximately 26.6% and 36.6% of participants reported being slightly to extremely unsatisfied with their office and home environments, respectively. Twenty-nine participants completed five-day studies, while one completed a three-day study due to contact lens issues.

The pre-screening survey covered university status, time spent in work locations, satisfaction with work locations, and prerequisites such as vision status. The onboarding survey included demographic information and sensitivity to Indoor Environmental Quality (IEQ) parameters.

The study leveraged the AirSpecs^9^ platform to collect local environmental and physiological data. The platform integrated various sensors configured at fixed sample rates and resolutions (see Table 2). Data were streamed via Bluetooth Low Energy (BLE) through a custom iOS application, allowing users to view real-time data and interact with micro-EMA surveys (see Fig. 2). All data were forwarded to an external server for monitoring by researchers to prevent data loss.

**Table 1:** 30 participants were selected from 23, 14, and 45 registrations at sites 1-3 based on responses to the pre-screening survey according to three criteria: (1) prioritizing graduate students, staff, and researchers who are likely to have work tasks that require concentration, (2) no need to wear glasses or can wear contact lenses to correct their vision, and (3) not extremely satisfied with all work environments.

PID	Age	Gender	Race/ethnicity	Occupation	Site	Work in built environment
1	24	Female	White	Master student	1	No
2	25	Female	White	University stuff	1	No
3	29	Non-Binary/third gender	Hispanic/Latinx	Master student	1	No
4	24	Male	Hispanic/Latinx, White	PhD student	1	No
5	26	Female	East Asian	University stuff	1	No
6	21	Male	White	Undergraduate student	1	No
7	22	Female	Asian-American	Undergraduate student	1	No
8	32	Male	East Asian	PhD student	1	Yes
9	21	Female	Hispanic/Latinx, Middle Eastern	Undergraduate student	1	No
10	24	Male	White	PhD student	2	No
11	46	Female	Hispanic/Latinx	Professor	1	Yes
12	31	Male	White	PhD student	2	No
13	27	Male	East Asian, White	PhD student	2	No
14	27	Male	Hispanic/Latinx, White	PhD student	2	No
15	30	Male	White	PhD student	2	No
16	45	Prefer not to say	White	Manager	2	No
17	33	Female	White	PhD student	2	No
18	52	Female	White	PhD student	2	No
19	27	Male	White	PhD student	2	Yes
20	25	Female	White	PhD student	2	No
21	23	Male	Southeast Asian	Undergraduate student	3	No
22	27	Female	East Asian	PhD student	3	Yes
23	23	Female	East Asian	Undergraduate student	3	No
24	24	Male	East Asian	PhD student	3	Yes
25	23	Female	East Asian	Master student	3	No
26	35	Male	South Asian	Master student	3	Yes
27	26	Female	East Asian	PhD student	3	No
28	24	Female	Southeast Asian	PhD student	3	Yes
29	23	Male	South Asian	Undergraduate student	3	No
30	29	Male	East Asian	Postdoc	3	Yes

**Table 2:** Summary of sensing parameters, their sampling settings, and corresponding locations on the AirSpecs device.

Parameter	Sensor	Sample rate	Accuracy	Location on glasses
Air temperature	SHT45	Every 5 sec	$\pm {0.1}^{\circ }{\rm{C}}$	Temple (right, outside as side-
Humidity			$\pm \mathrm{1.0 \% }$	board), bridge (front)
VOC	SGP41	Every 5 sec	$\pm \mathrm{15 \% }$	Temple (right, outside as side-
NOx			$\pm 50{\rm{ppb}}$	board), bridge (front)
Iluminance (lux)	TSL27721	Every 1 sec	—	Bridge (front)
Spectrum	AS7341	Every 5 sec	—	Bridge (front)
IAQ	BME688	Every 5 sec	$\pm \mathrm{15 \% }$	Bridge (front)
(e)${{\rm{CO}}}_{2}$			$\pm \mathrm{15 \% }$	
Noise (dBA)	ICS-43434	48000 Hz	—	Temple (left, outside)
Audio Frequency		(activate 85 ms every min)	—	
Skin temperature	TPIS 1S 1385	Every 1 sec	$\pm {0.3}^{\circ }{\rm{C}}$	Temple (right, inside), bridge (back), nose pad (right)
Blink	QRE1113	1000 Hz	—	Nose pad (left)

**Fig. 3:**
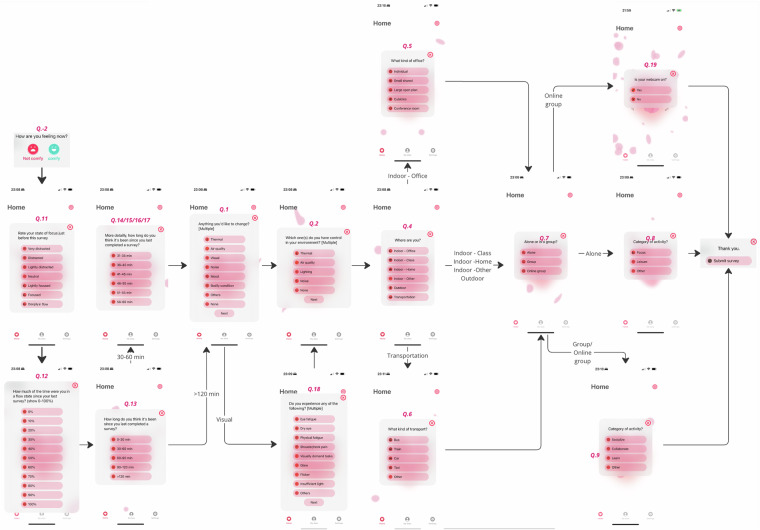
The question flow is designed based on the micro-ecological momentary assessment (micro-EMA) question flows for building occupants’ experience of space^14^.

To prompt users to take surveys, peripheral cues were employed to reduce disruption to natural experiences. Inspired by Ramsay and Paradiso’s work on using a slowly changing peripheral LED as a secondary task^11^, the study utilized LEDs built into AirSpecs to signal survey times at random intervals between 1 and 1.5 hours. The LED gradually transitioned from blue to green over 53 seconds to indicate survey availability (see Fig. 3). If users did not respond within 15 minutes, a vibration notification was sent to their Apple Watch. Once users engaged with the micro-EMA survey, the LED reverted to blue until the next survey interval. The micro-EMA survey focused on introspective aspects, querying perceived focus level, time perception related to flow states, current context, and sources of discomfort.

## Data Records

The dataset has been uploaded to Figshare and includes all data sources, including metadata^12^. The data records collected from four sources (AirSpecs, AirSpecs App, Cozie App, and Empatica E4) are synchronized using a UTC timestamp and a unique participant ID assigned to each participant. The participants’ experiment schedule, along with pre-screening, demographic information, exit surveys, and open coding of sensor rearrangement co-design, are included in this dataset (participants.csv and consolidated_atlas.csv). The meanings of the columns in the consolidated data, as well as the columns in the environmental and physiological data files, can be found in Summary_of_derived_data.xlsx, under the “column meanings” tab. The consolidated_atlas.csv includes answers to exit interview questions, which are documented in Exit_survey_and_interview_questions.pdf in the repository.

The details of the raw data and pre-processed consolidated data frames for each source are as follows:

### Environmental and physiological sensing data around the user’s face from AirSpecs

The raw sensing data were exported from InfluxDB and stored in CSV format, and were consolidated into data frames in pickle format per sensor name and location. All of these data frames share timestamps, a unique participant ID, a phone ID (reflecting the connected glasses), and the experiment location. The rest of the columns reflect the environmental and physiological parameters recorded (e.g., ambient temperature, humidity, and skin temperature). The consolidated data is compressed within AirSpec_data.7z.

### EMA from AirSpecs App

We aimed to capture users’ intuitive comfort perceptions using our Ecological Momentary Assessment (EMA) questionnaire. The questionnaire automatically progressed to the next question for single-answer queries (up to 11 questions), and participants were instructed to click “next” after completing multiple-choice questions (maximum of 3 questions). Recognizing that participants might be engaged in conversations or otherwise occupied, we allowed them to answer only the initial question on their comfort state (e.g., comfy vs. not comfy) to record reaction times accurately. Post-experimental interviews indicated that participants could complete the remaining questionnaire within 5 minutes, so we grouped these delayed responses with their initial reaction times if submitted within this timeframe. Otherwise, these responses were treated as voluntary additions.

The EMA is always initiated from the comfort state question triggered by an Apple Watch wrist-up event or iOS icon click, enabling spontaneous reaction time recording upon selecting a comfort state. User interactions with the app interfaces were logged to gauge interest in Indoor Environmental Quality (IEQ) dimensions. In our consolidated dataset, timestamps and corresponding clicks were matched with EMA, and reaction time data was recorded within a 10-minute window.

We recommend working with the consolidated data due to the complexity of interpreting raw data without familiarity with the app’s architecture and data transmission protocols.

We collected 1,175 micro-EMA surveys with associated reaction times across 30 participants across three sites: 352 from Site 1, 491 from Site 2, and 332 from Site 3. The average number of surveys per participant was $39.2\pm 15.6$ (mean ± SD). Surveys were predominantly completed via the Apple Watch application (1,004) compared to the phone application (161). The results of the survey data across participants is stored in survey_reactionTime_uiClick.csv.

### Activity and physiological data at the user’s non-dominant hand from Cozie App

When worn on the non-dominant wrist, the Apple Watch facilitated effortless navigation using the dominant hand. Participants were instructed to wear the Apple Watch on their non-dominant hand and the Empatica E4 on their dominant hand. The Cozie App, being open-source, integrates its EMA function into the AirSpecs App described earlier, eliminating the need for participants to switch between apps during the experiment. Meanwhile, the Cozie App continued to run in the background, fetching Apple HealthKit data recorded by the Apple Watch and iPhone. Detailed parameters retrieved from HealthKit are outlined in the Cozie App’s documentation available at https://cozie-apple.app/docs/download_data/data_overview. The data from the Cozie App is stored in pickle format (cozie.pkl) and can be deserialized using the Python pickle module.

### Physiological data at the user’s dominant hand from Empatica E4

Empatica E4 data were stored locally on the wristband for five days, and we downloaded original CSV files per sensing parameter per Empatica E4 session ID using its official app. To link session IDs with participants, we pre-processed these raw E4 files, aggregating all sensing parameters per participant per session based on their experiment schedule. These data can be further synchronized with other sources using unique participant IDs and timestamps. The data is compressed within E4_formatted.7z.

## Discontinuity

During the experiment, we experienced a data storage failure that resulted in the loss of physiological data from AirSpecs for Site 2. We did not institute an automatic data backup system because of the potential additional load it would have put on our server during data collection. The error was the result of a default InfluxDB data retention policy of 3 months that the authors were not aware of. Site 1’s data was backed up to an external AWS server before beginning work at Site 2 but that data wasn’t backed up until the collection at Site 3 was complete. However, survey data, as well as Empatica E4 and Apple Watch data, were preserved for all sites. To mitigate this issue for future experiments, the retention policy should be set to a range more appropriate for the experiment and data should be backed up automatically during off-peak hours or manually on a more periodic basis. We also do not have the blink data from Site 1 due to an issue with the initial firmware on the AirSpecs glasses that was resolved in an update prior to starting the other sites. The initial firmware did not fully power on the blink detection sensor in low- to medium-light conditions (e.g., indoor environments) which resulted in a sensor signal with limited visibility of blink occurrences. These issues causing the discontinuity have been fixed after the study in this paper for potential future studies with other researchers using the AirSpecs system.

For the Cozie data, we don’t have data for P8 and P30, likely due to the Cozie App being accidentally shut off by the participants for the duration of the experiment. The Empatica E4 dataset also doesn’t include data for P23 and P26 for reasons unknown but likely hardware failures.

## Technical Validation

The accuracy of individual environmental sensors (part numbers listed in Table 2) can be found in their respective data sheets on the manufacturers’ websites. We validated the non-contact skin temperature sensors by recording 30 consecutive measurements after stabilization and comparing them against the reference sensor, iButton® temperature loggers DS1922L (MAXIM Integrated, US) mounted at the same location (Table 3). The iButton skin temperature sensors were calibrated at the Laboratory of Integrated Comfort Engineering (ICE), École Polytechnique Fédérale de Lausanne, using a Julabo CORIO CD water bath and a precision thermometer with an uncertainty of 0.015 °C. The calibration resulted in an accuracy of ±0.2 °C for the iButtons^13^. Since we could not use both the reference sensor and the non-contact sensor simultaneously, we took a series of measurements alternating between the two in quick succession. We found that the average difference between the reference sensor and ours for the temple location was negligible, likely due to the large, relatively flat surface of the temple providing ideal conditions for the optical temperature sensors. The nose locations showed a larger temperature offset between the reference and our non-contact sensors, but the standard deviation was nearly the same as for the temple locations. This indicates that, aside from requiring a temperature offset, the performance should be similar across temperature sensors.

**Table 3:** Average difference of non-contact skin temperature measurements using calibrated thermocouple as reference (n = 30).

Thermopile Location	Average (°C)	Standard Deviation
Nose Tip	0.78	0.43
Nose Bridge	1.05	0.57
Temple (Front)	−0.20	0.40
Temple (Mid)	−0.10	0.38
Temple (Rear)	0.11	0.39

## Usage Notes

Within our data, there is variability in the length of sensor collections for each participant each day and in any discontinuities throughout a given day. This variability is due to the study being unsupervised by researchers, allowing participants to remove the sensors when performing tasks that might damage the units (e.g., swimming) or disrupt their activities. When using the data, discontinuities should be taken into consideration, and interpolation should be approached with caution. While some physiological and environmental parameters generally change slowly (e.g., face temperature, ambient temperature), others can vary abruptly (e.g., gases).

We have both quantitative and qualitative data that can be used to experiment with personalized model building and determine which sensing modalities and locations are the most optimal for a particular objective function. Researchers can also use the recorded response delay of the survey and results from prior work on relating response delay to focus levels to build more intelligent comfort models that consider human internal states^11^.

We did not perform further validation of the sensor subsystems beyond what was provided by the manufacturer. The AirSpec systems were all constructed for the study in the months prior, using new batches (late 2022) of sensors procured from the manufacturer. Given that this experiment was conducted in uncontrolled environments, we cannot guarantee the proportional contribution of an individual’s environment to their own thermodynamic microclimate. This is influenced by both the individual’s physiological responses and the environment they are in (e.g., ventilation levels). For instance, if a person is sweating and/or breathing heavily in a room with low airflow, the humidity around their face is expected to increase. Thus, a humidity sensor mounted on a pair of eyeglasses will record a combination of the environmental humidity level and the individual’s contribution to it. The participants were informed on the potential difference between measurement from the glasses and other ambient environmental sensing devices with a note on the *Settings* screen of the App in Fig. 2 to minimize the impact of sensor value ranges on the subjective reflection during EMA.

We also did not measure any potential sensor drift between sites, as all studies across the three locations were conducted within a continuous five-month period. However, no manufacturer datasheets report long-term drift except for the SHT45 temperature and humidity sensors, where the drift is less than 0.2% relative humidity per year. If an additional study is conducted to measure long-term comfort, it is recommended to calibrate the sensors more regularly to compensate for any potential drift in absolute readings or sensitivities.

The total sample size of the study was 30 participants, evenly distributed across three cities in distinctly different geographic regions. Singapore is generally more tropical year-round, with higher humidity and temperature compared to the other two cities, while Fribourg typically experiences the mildest temperature and humidity ranges during the summer months. In a tropical region such as Singapore, it was particularly interesting to understand user comfort as people experience significant temperature and humidity fluctuations when entering or exiting buildings, as opposed to climatic regions where such variations are less pronounced (e.g., Fribourg).

A goal of this study was to conduct a limited exploration in each of these cities using a variety of collection mechanisms to identify potential failure modes when scaling to larger populations or longer durations. The participants were limited to university students and staff due to the access to participant pools. Therefore, the results are not representative of the larger populations in each region nor are they indicative of long-term comfort variations. However, these data can be used to understand short-term comfort and its relation to individuals’ perceptions, as well as to support a preliminary analysis of variations across geographical regions. Nevertheless, a larger study with a wider demographic is warranted for more generalizable results.

There are no access restrictions or no limitations on data use for our collected dataset.

## Supplementary information

Supplementary Information

## Data Availability

The AirSpec firmware code to reference specific sensor settings and system architectures can be found here: https://github.com/pchwalek/airspec. The iOS application with survey implementation can be found here: https://github.com/sailinz/AirSpec_iOS.
